# Evaluation of Structurally Distorted Split GFP Fluorescent Sensors for Cell-Based Detection of Viral Proteolytic Activity

**DOI:** 10.3390/s21010024

**Published:** 2020-12-23

**Authors:** Miguel R. Guerreiro, Ana R. Fernandes, Ana S. Coroadinha

**Affiliations:** 1 iBET, Instituto de Biologia Experimental e Tecnológica, Apartado 12, 2781-901 Oeiras, Portugal; mguerreiro@ibet.pt (M.R.G.); ana.fernandes@ibet.pt (A.R.F.); 2 Instituto de Tecnologia Química e Biológica António Xavier, Universidade Nova de Lisboa, Av. da República, 2780-157 Oeiras, Portugal

**Keywords:** cell-based assay, fluorescent biosensor, structural distortion, intein, eglin c, coiled-coils, protease, adenovirus, lentivirus

## Abstract

Cell-based assays are essential for virus functional characterization in fundamental and applied research. Overcoming the limitations of virus-labelling strategies while allowing functional assessment of critical viral enzymes, virus-induced cell-based biosensors constitute a powerful approach. Herein, we designed and characterized different cell-based switch-on split GFP sensors reporting viral proteolytic activity and virus infection. Crucial to these sensors is the effective—yet reversible—fluorescence off-state, through protein distortion. For that, single (protein embedment or intein-mediated cyclization) or dual (coiled-coils) distortion schemes prevent split GFP self-assembly, until virus-promoted proteolysis of a cleavable sequence. All strategies showed their applicability in detecting viral proteolysis, although with different efficiencies depending on the protease. While for tobacco etch virus protease the best performing sensor was based on coiled-coils (signal-to-noise ratio, SNR, 97), for adenovirus and lentivirus proteases it was based on GFP11 cyclization (SNR 3.5) or GFP11 embedment distortion (SNR 6.0), respectively. When stably expressed, the sensors allowed live cell biosensing of adenovirus infection, with sensor fluorescence activation 24 h post-infection. The structural distortions herein studied are highly valuable in the development of cellular biosensing platforms. Additionally highlighted, selection of the best performing strategy is highly dependent on the unique properties of each viral protease.

## 1. Introduction

The growth of human population, urbanization, and long distance travelling have contributed markedly to the emergence of pathogenic virus outbreaks of unprecedented size and geographical extension [1]. For many viral infections, antiviral drugs targeted to block virus entry in host cells or to block viral enzymes crucial to virus replication—such as the proteases—constitute the only therapeutic approach to reduce virus load in infected patients and decrease the development of chronic disease [2]. In parallel, the last years also witnessed an explosion of new virus-based biopharmaceuticals. While traditional vaccination schemes have historically received most of the attention [3], the interest in viral vectors is growing remarkably, whether be adenoviruses for oncolytic virotherapies [4] or lentiviral vectors for chimeric antigen receptor T-cell therapies [5].

To support fundamental and applied research in virology, development of virus-based biopharmaceuticals, and screening of antivirals, cell-based assays are of outmost importance for functional characterization of viral enzymes and viruses. Regarding the latter, despite viral plaque and colony forming assays [6,7] being considered the “gold standards” for virus detection and quantification, they are time-consuming and lack high-throughput. Faster and easier to perform methods often rely on viral genome coupled reporter genes such as Green fluorescent protein (GFP). However, virus labelling strategies may impact negatively on virus production and its genetic stability [8,9].

Virus-induced cell-based biosensors constitute a powerful approach, overcoming the limitations of using virus-labelling strategies while additionally allowing functional assessment of critical viral enzymes. Essential to the virus life cycle, viral proteases cleave viral polyprotein precursors—for virus maturation [10]—and host cell proteins—for inhibition of host protein translation [11] or to bypass innate immune responses [12]. As such, viral proteases constitute important targets not only for antiviral drug screening [13] but also as key elements to diagnose and report virus infection. For such protease-sensing systems, fluorescent proteins such as split GFP [14] are highly advantageous. In this version, one fragment of the split GFP contains 10 β-strands (GFP10), where the three residues forming the chromophore remain almost nonfluorescent until complementation. The other fragment contains the 11th β-strand of GFP (GFP11), where the highly conserved Glu222 catalyzes chromophore maturation after rapid self-assembly with GFP10 fragment. A number of split GFP sensors dependent on viral protease activity have been developed [15,16]. Similar strategies were also applied for detection of cellular proteases [17,18]. However, those methodologies require laborious fluorescence analysis, lack throughput, or were not assessed neither in mammalian cells nor, importantly, in response to virus infection.

By combining the high specificity of virus host cells with optical detection of genetically encoded and conditionally activated switch-on fluorescent sensors, we have designed, optimized, and characterized innovative cell-based assays to detect viral proteolytic activity and label-free virus infection. Structural distortion—herein referred as holding a peptide or a protein in a non-native structural conformation—of one (by GFP11 embedment or cyclization) or both (GFP10 and GFP11 with heterodimerizing coiled-coils) of the split GFP fragments prevents their self-assembly until a cleavable sequence—acting as a switch—is processed by the viral protease. The different strategies of structural distortion were herein assessed in response to the highly specific tobacco etch virus protease and the clinically important adenoviral and lentiviral proteases. While all strategies showed to be valid in the development of virus protease-dependent sensors, they performed differently for the proteases under study due to the intrinsic properties of each protease. As shown in transient screenings, for tobacco etch virus protease the best performing sensor was based on coiled-coils (signal-to-noise ratio, SNR, 97), while for adenovirus and lentivirus proteases it was based on GFP11 cyclization (SNR 3.5) or GFP11 embedment distortion (SNR 6.0), respectively. Moreover, stable expression in mammalian cells of these genetically encoded sensors enabled establishment of a biosensing platform for monitorization of adenovirus infection, with sensor fluorescence activation 24 h post-infection.

## 2. Materials and Methods

### 2.1. Plasmids

The plasmid pRRLSIN.cPPT.PGK-GFP.WPRE (Addgene plasmid No. 12252, kindly provided by Didier Trono through the Addgene plasmid repository, Watertown, MA, USA) was modified to substitute the human phosphoglycerate kinase 1 promoter and GFP by a cytomegalovirus promoter driving the expression of GFP10 (Sandia Biotech Inc, Albuquerque, NM, USA), and an encephalomyocarditis virus internal ribosomal entry site (amplified from pIRESGALEO [19]) driving the expression of *Sh ble* (amplified from pMONO-zeo-mcs; Invivogen, San Diego, CA, USA) or *pac* genes (amplified from pSELECT-puro; Invivogen).

By replacing GFP10, the plasmids described above served as backbones for the sensor (structurally distorted GFP10 or GFP11) and protease constructs. Briefly, for the GFP11 embedment strategy—e11 chimeras—the internal loop of eglin c (Ser42 to Arg49) [15] was replaced by GFP11 and the ENLYFQ*S cleavable sequence (recognized by the tobacco etch virus protease, TEVp; asterisk denoting scissile bond) [20]—e11-ENLYFQS sensor (Table S1). In the GFP11 cyclization strategy—cy11 chimeras—C- and N-intein fragments of the *Nostoc punctiforme* DnaE split intein (*Npu* DnaE) [21] were linked, respectively, to the N- and C-termini of GFP11 in fusion with ENLYFQ*S—cy11-ENLYFQS sensor (Table S2). In the coiled-coil strategy—cc10/11 chimeras—E5/K5 heterodimerizing coiled-coils were used [18]. E5 was fused N-terminal to GFP10, while ENLYFQ*S and K5 were fused C-terminal—cc10-ENLYFQS sensor (Table S3). Inversely, K5 was fused N-terminal to GFP11, while ENLYFQ*S and E5 were fused C-terminal—cc11-ENLYFQS sensor (Table S4). Direct replacement of ENLYFQ*S cleavable sequence by LRGA*G [22], IVGL*G and EEGE*G [23] (for the adenovirus protease, AVP), or GIF*LET, GSGIF*LETSL, and IRKIL*FLDG [24,25] (for the human immunodeficiency virus 1 protease, HIV-1 PR) by site-directed PCR mutation created the “.v0” chimeras of the corresponding sensors. Addition of one (.v1) or two (.v2) glycine spacers surrounding each side of the cleavable sequence of the sensors described above was also performed by site-directed PCR mutation. Constructs coding for the viral proteases were also designed: AVP in fusion with a 11-residue peptide from the C-terminus of precursor protein VI (pVIc) with a MVGL*G cleavable sequence [26]; and the stable S219V variant of TEVp [27].

All constructs were generated using custom DNA oligonucleotides, gBlocks Gene Fragments (Integrated DNA Technologies, Inc., Coralville, IA, USA), custom gene synthesis (GenScript, Piscataway, NJ, USA), standard molecular biology techniques, In-Fusion HD Cloning (Takara Bio Inc., Mountain View, CA, USA), and confirmed by extensive sequencing of the cloned fragments. Further details on the developed sensor constructs are provided in Table S5.

### 2.2. Mammalian Cell Lines

293 [HEK-293] (ATCC CRL-1573), 293T (ATCC CRL-3216), and 293 FLEX S11 [28] were routinely cultivated in Dulbecco’s modified Eagle’s medium (DMEM; Gibco, Paisley, UK), supplemented with 10% (*v*/*v*) of fetal bovine serum (Gibco), and maintained at 37 °C in an incubator with humidified atmosphere of 5% CO_2_ in air.

### 2.3. 293 Cell Transduction and Selection

Establishment of 293 sensor cells was accomplished by lentiviral transduction. First, 293T cells plated at 8 × 10^4^ cells/cm^2^ the day before were transiently co-transfected using linear 25 kDa poly(ethylenimine) (PEI; Polysciences Inc., Hirschberg, Germany) with the developed pRRLSIN plasmids—coding for GFP10 fragment, e11 or cy11 sensors—and pMDLg/pRRE (Addgene plasmid No. 12251), pMD2.G (Addgene plasmid No. 12259) and pRSV-Rev (Addgene plasmid No. 12253) [29]. Viral supernatants were collected 48 h later, clarified by 0.45 µm filtration, and stored at −80 °C. 293 cells, plated at 8 × 10^4^ cells/cm^2^ the day before, were transduced with lentivirus coding for GFP10 (previously titrated in 293 FLEX S11 cells by GFP transcomplementation titration [28]) at a multiplicity of infection of 5 in presence of 8 µg/mL Polybrene (Sigma-Aldrich, St. Louis, MO, USA). After 48 h, cells were selected using 200 µg/mL Zeocin (Invivogen). This selected 293 GFP10 population was then transduced with lentiviral stocks coding for e11 or cy11 sensors, further selected using 1.5 µg/mL Puromycin (Invivogen), and the resulting populations characterized.

### 2.4. Adenoviral Vector Stocks

Label-free human recombinant E1-deleted adenovirus serotype 5 (AdV; provided by Dr. Geneviève Libeau, CIRAD-EMVT, Montpellier, France) were amplified, purified and stored as previously described [30]. AdV stocks were quantified by tissue culture infectious dose 50 assay in 293 cells according to the method of Spearman and Kärber, described in Darling et al. [31].

### 2.5. Split Sensors Characterization

For initial screenings, transient transfections were performed. Briefly, 293T cells plated at 8 × 10^4^ cells/cm^2^ the day before were co-transfected with 5 µg of total DNA/(10^6^ cells) using PEI with each of the sensor-coding plasmids (and GFP10-coding plasmid where needed for complementation), and either TEVp-coding plasmid, AVP-coding plasmid, HIV-1 PR-coding plasmid (psPAX2, Addgene plasmid No. 12260) or a mock plasmid.

For the assessment of stable sensors cells, 293 GFP10 cells stably expressing either e11 or cy11 optimal sensors were seeded at 1 × 10^5^ cells/cm^2^. In the next day, the culture medium was removed, and cells were infected with AdV at a multiplicity of infection of 5, in 0.2 mL of nonsupplemented DMEM (Gibco). After 1 h, 0.3 mL of fresh supplemented DMEM was added.

Forty-eight hours post-transfection or at each post-infection time point, cells were: (i) observed by fluorescence microscopy (DMI6000, Leica, Wetzlar, Germany) at 100× optical magnification using a 488 nm laser line, a BP480/40 nm excitation filter, and a BP527/30 nm emission filter; (ii) assessed by flow cytometry (CyFlow Space, Sysmex Partec GmbH, Görlitz, Germany) with gates set using nontransfected 293T cells as negative control, and the geometric mean GFP fluorescence intensity of GFP positive cells measured within the positive gate.

### 2.6. Evaluation of GFP10 and GFP11 Levels in 293 Sensor Cells

To assess the levels of GFP10 available for transcomplementation, 293 GFP10 population and 293 e11 and 293 cy11 sensor cells were seeded at 6 × 10^4^ cells/cm^2^. After 24 h, cells were transduced with a characterized stock of retrovirus harboring a nondistorted version of GFP11 fragment (produced and characterized as described in [28]) in presence of 8 µg/mL Polybrene (Sigma-Aldrich). After 48 h, control (nontransduced) and transduced cells were analyzed by flow cytometry for geometric mean GFP fluorescence intensity.

To determine relative transgene copy number integration (whether coding for e11 or cy11 sensors), genomic DNA from parental 293 GFP10 population, and from 293 e11 and 293 cy11 sensor cells was extracted using DNeasy Blood & Tissue Kit (Qiagen, Valencia, CA, USA). Quantitative PCR was performed using LightCycler 480 SYBR Green I Master PCR kit (Roche Applied Science, Mannheim, Germany) and primers for the Woodchuck hepatitis virus post-transcriptional regulatory element (*WPRE*) (Table S6) on a LightCycler 480 Real-Time PCR System (Roche Applied Science). The number of copies per cell was quantified relatively to 293 GFP10 parental cells, after normalization to *RPL22* reference gene, using the 2^−ΔΔCT^ method [32].

### 2.7. Data and Statistical Analysis

SNR was used to evaluate the performance of the developed sensors. Herein, SNR refers to the ratio of total accumulated GFP fluorescence (geometric mean fluorescence of GFP positive cells times GFP positive scored events, as measured by flow cytometry) upon sensor activation (signal), to total accumulated GFP fluorescence of mock or noninfected controls (noise). Statistical analysis was performed using unpaired, two-tailed Student’s *t*-test or one-way analysis of variance (ANOVA) followed by Tukey’s post-hoc test. *p* values < 0.05 were considered statistically significant.

## 3. Results

### 3.1. Design of Switch-On Split GFP Sensors Activated by Proteolysis

**Scheme 1:**
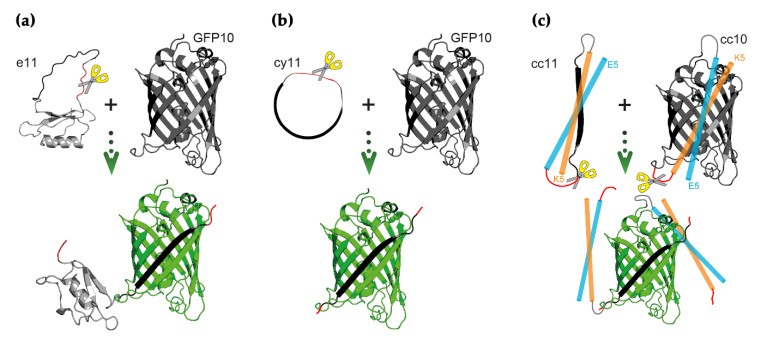
Schematic illustrations of the switch-on split fluorescent proteins activated by proteolysis. (**a**) GFP11 fragment is embedded as a surface loop on the small protein eglin c—e11 strategy. (**b**) GFP11 fragment is cyclized by *Nostoc punctiforme* DnaE split intein—cy11 strategy. (**c**) GFP10 and GFP11 fragments are flanked by α-helical heterodimeric E5/K5 coiled-coils—cc10/11 strategy. All three strategies lead to structural distortion—of one or both—of split GFP fragments, with concomitant inhibition of fragment self-assembly. Proteolytic cleavage at the cleavable sequence (scissor representing the protease) leads to relief of structural distortion and fluorescence emission. Illustrative structures were adapted from Protein Data Bank accession codes 1GFL (GFP, GFP10, and GFP11) and 1ACB (eglin c), and are not at scale. For simplicity, the α-helical E5/K5 coiled-coils are depicted as straight cylinders.

For the establishment of split fluorescent sensors whose self-assembly is controlled by proteolytic activity, three strategies of structural distortion of split GFP fragments were developed, characterized, and evaluated (Scheme 1).

In native GFP, the GFP11 fragment is an elongated β-strand comprised of 16 residues and more than 40 Å [33]. It can be hypothesized that by holding GFP11 in a non-native conformational state, its self-assembly tendency towards GFP10 and consequent catalysis of GFP chromophore maturation is impaired. As such, in one of the approaches, GFP11 peptide in fusion with a cleavable sequence was embedded as a surface loop on the small protein eglin c [15]—hereafter referred as e11 strategy (Scheme 1a). This anchorage to the thermodynamically stable backbone of eglin c can lead GFP11 to acquire a bent conformation—instead of an elongated one—with the N- and C-termini brought closer in space (to less than 40 Å) [34]. This noncovalent single distortion strategy was compared to introducing the distortion into GFP11 termini via covalent ligation. Herein, protein splicing promoted by the efficient *Npu* DnaE split intein creates cyclized chimeras where GFP11 termini are covalently linked by a protease cleavable sequence—cy11 strategy (Scheme 1b). To further prevent self-assembly of GFP fragments before proteolysis, a dual distortion scheme where both GFP10 and GFP11 fragments are flanked with heterodimerizing E5/K5 coiled-coils (in fusion with a cleavable linker) [18] was also assessed—cc10/11 strategy (Scheme 1c). In all three strategies structural distortion prevents GFP fragments self-assembly until proteolytic cleavage takes place at the cleavable sequence, thereby allowing fragment complementation and fluorescence emission.

**Figure 1:**
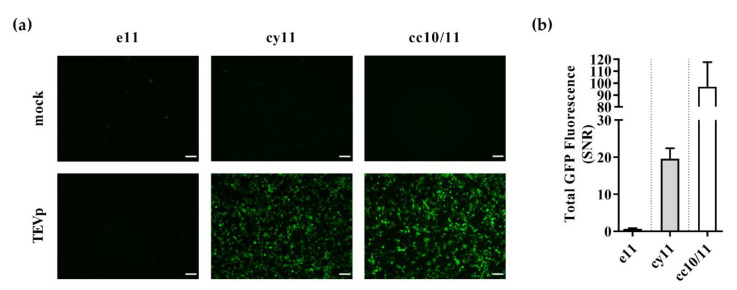
Validation of embedment, cyclization, and coiled-coil sensing strategies for detection of tobacco etch virus proteolytic activity. 293T cells were transiently co-transfected with plasmids coding for either embedded GFP11 (e11), cyclized GFP11 (cy11) or coiled-coil GFP10 and GFP11 (cc10/11) sensors, GFP10-coding plasmid where needed for complementation, and either a mock plasmid or tobacco etch virus protease (TEVp) coding plasmid. After 48 h, (**a**) fluorescence microscopy images were acquired, and (**b**) sensor performance as given by GFP signal-to-noise ratio (SNR) was assessed by flow cytometry. Scale bar = 100 µm. Data shown as mean ± SD of at least three independent experiments.

To assess the conditional activation of the proposed designs, sensors containing the ENLYFQ*S residues recognized by the highly specific and active TEVp were evaluated. For this proof-of-concept, 293T cells were transiently co-transfected with plasmids coding for either e11, cy11 or cc10/11 chimeras, GFP10 (when needed for complementation), and a mock or TEVp. All strategies were effective on preventing split GFP fragments self-assembly before proteolytic cleavage, as shown by the lack of fluorescence emission (Figure 1a, top panel).

In the presence of TEVp (Figure 1a, bottom panel, and Figure S1), while surprisingly e11 strategy showed no fluorescence activation, both cy11 and cc10/11 strategies showed effective activation of GFP fluorescence. Flow cytometry analysis confirmed the fluorescence microscopy imaging observations, with a total GFP SNR of 0.6 ± 0.2 (e11 strategy), 20 ± 3 (cy11 strategy), and 97 ± 21 (cc10/11 strategy) (Figure 1b and Figure S1).

Together, these results confirmed structural distortion of split GFP fragments as a valid approach towards mammalian cell-based switch-on split GFP sensors activated by proteolysis.

### 3.2. Switch-On Sensors Activated by Adenovirus and Lentivirus Proteases

**Figure 2:**
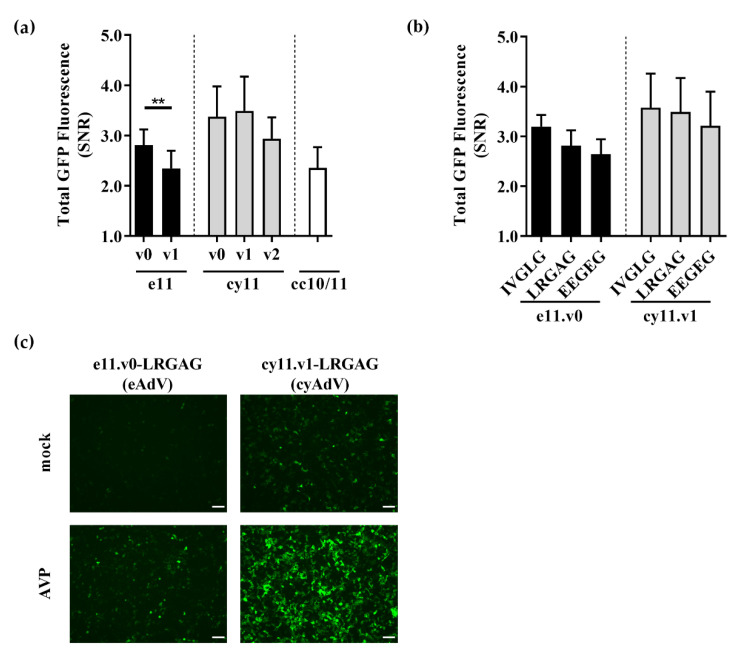
Evaluation of the different sensors and the impact of backbones and cleavable sequences on sensor performance for detection of adenoviral proteolytic activity. Sensor performance as given by GFP signal-to-noise ratio (SNR) of (**a**) different backbones for e11, cy11, and cc10/11 strategies with a LRGA*G cleavable sequence, and (**b**) different cleavable sequences in the optimal e11.v0 and cy11.v1 backbones were assessed by flow cytometry 48 h after transient co-transfection. Data shown as mean ± SD of at least three independent experiments. **, *p* < 0.01 as given by an unpaired, two-tailed Students’ *t*-test. (**c**) Fluorescence microscopy of e11.v0-LRGAG (eAdV) and cy11.v1-LRGAG (cyAdV) sensors 48 h after co-transfection with GFP10 and a mock plasmid or AVP-coding plasmid. Scale bar = 100 µm.

The applicability of these strategies to the clinically important adenovirus and lentivirus proteases was then assessed. Firstly, the cleavable sequences in TEVp sensors were substituted by the LRGA*G cleavable sequence detected by the AVP. Transient co-transfection of 293T cells showed an increase of total GFP fluorescence over the negative control in all three strategies, suggesting sensor activation upon proteolysis: SNR, 2.8 ± 0.3 (e11.v0 sensor); SNR, 3.4 ± 0.6 (cy11.v0 sensor); and SNR, 2.4 ± 0.4 (c10/11 sensors); Figure 2a and Figure S2–S4.

The two best performing strategies—e11 and cy11—were then further characterized. In the initial sensor backbones—e11.v0 and cy11.v0—glycine spacers surrounding LRGA*G residues were sequentially added to evaluate a potential increase in steric freedom and exposure of the cleavable sequence to AVP. In the e11 strategy, addition of one glycine spacer (e11.v1) led to a significant reduction in SNR performance (SNR, 2.3 ± 0.4; Figure 2a). As for cy11 strategy, addition of one (cy11.v1) or two (cy11.v2) glycine spacers did not significantly change SNR performance (as given by ANOVA analysis followed by Tukey’s post-hoc test), despite a clear impact in fluorescence emission (Figure 2a and Figure S3). Taken together, backbones e11.v0 (SNR, 2.8 ± 0.3) and cy11.v1 (SNR, 3.5 ± 0.7) were chosen as the optimal backbones for AVP activity detection.

Different cleavable sequences may be recognized differently by the AVP [22,35,36] and introduce different levels of structural distortion. As such, cleavable sequences naturally occurring in the adenoviral precursor proteins—IVGL*G and EEGE*G—were assessed in the optimal backbones. As shown in Figure 2b, no significant impact in SNR performance was observed (as given by ANOVA analysis followed by Tukey’s post-hoc test, within each strategy), despite affecting fluorescence emission levels (Figures S2 and S3). In common to both strategies, IVGL*G-containing sensors presented a marked decrease in fluorescence emission, whereas EEGE*G-containing sensors presented increased fluorescence emission at the expense of slightly lower SNR performance (Figures S2 and S3). Overall, these results showed that sensors e11.v0-LRGAG and cy11.v1-LRGAG—hereafter referred for simplicity as eAdV and cyAdV, respectively—were the best performing switch-on split fluorescent sensors for detection of AVP activity (Figure 2c).

**Figure 3:**
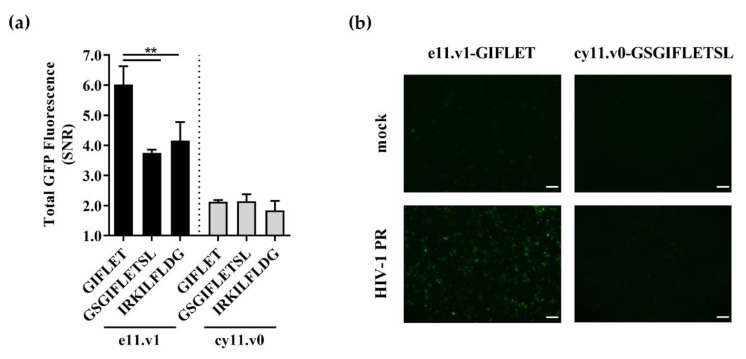
Impact of backbones and cleavable sequences on e11 and cy11 sensors performance for detection of lentiviral proteolytic activity. (**a**) Sensor performance as given by GFP signal-to-noise ratio (SNR) of different cleavable sequences in e11.v1 and cy11.v0 backbones was assessed by flow cytometry 48 h after transient co-transfection. Data shown as mean ± SD of at least three independent experiments. **, *p* < 0.01 as given by ANOVA followed by Tukey’s post-hoc test. (**b**) Fluorescence microscopy of e11.v1-GIFLET and cy11.v0-GSGIFLETSL sensors 48 h after co-transfection with GFP10 and a mock plasmid or HIV-1 PR-coding plasmid. Scale bar = 100 µm.

A similar optimization approach was followed for the detection of HIV-1 PR activity. For that, different sensor backbones and/or cleavable sequences—the non-natural GIF*LET and GSGIF*LETSL, and the naturally occurring IRKIL*FLDG—were evaluated. Again, an increase in fluorescence emission was observed when sensors were transiently co-transfected with HIV-1 PR into 293T cells (Figures S5 and S6). For the detection of HIV-1 protease activity, addition of a glycine spacer in e11 chimera increased its performance (e11.v1-GIFLET with SNR of 6.0 ± 0.6; Figure S5b) and no cleavable sequence was better than GIF*LET (Figure 3). A similar pattern was observed when using the non-optimal e11.v0 backbone (Figure S5c). In contrast to e11 strategy, cy11 sensors showed low SNR performances, with the best performing sensor (cy11.v0-GSGIFLETSL) only reaching a SNR of 2.1 ± 0.2 (Figure 3 and Figure S6).

As showed for AVP and HIV-1 PR, the assessed switch-on sensors showed potential for mammalian cell-based biosensing platforms for the detection of viral proteolytic activity.

### 3.3. Whole Cell Biosensing Platform for Monitoring of Adenoviral Infection

**Figure 4:**
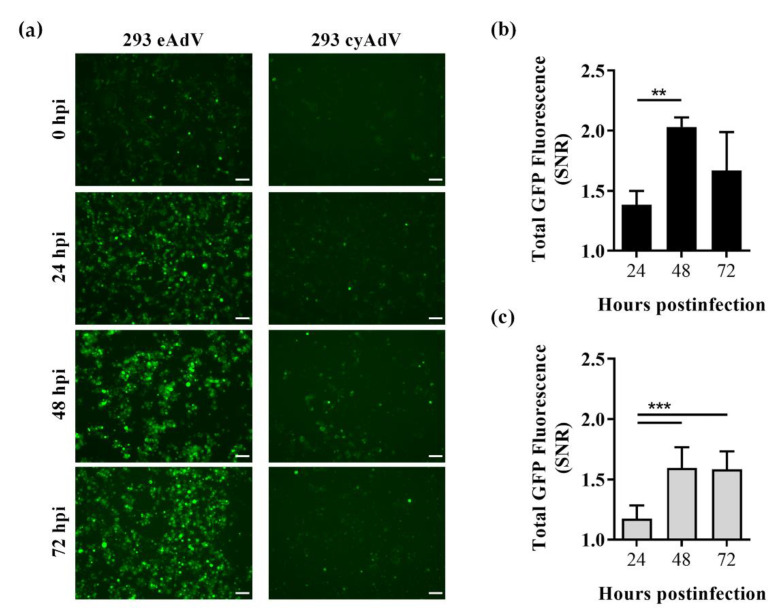
Characterization of whole cell biosensing platforms in response to adenoviral infection. 293 GFP10 cells stably expressing eAdV or cyAdV sensors were infected with adenoviral vectors at a multiplicity of infection of 5. At the indicated time points post-infection, sensor cells were (**a**) visualized by fluorescence microscopy, and GFP signal-to-noise ratio (SNR) was evaluated by flow cytometry for performance of (**b**) eAdV or (**c**) cyAdV sensors. Scale bar = 100 µm. Data shown as mean ± SD of at least three biological replicates. **, *p* < 0.01; ***, *p* < 0.001, as given by ANOVA followed by Tukey’s post-hoc test.

To assess the applicability of the structural distortion strategies as virus-induced cell-based biosensors, the optimal eAdV or cyAdV sensors (and GFP10 fragment) were stably expressed in 293 cells. When infected with label-free AdV, sensor cells had their GFP fluorescence intensity increased throughout infection time (Figure 4a), and already significantly at 24 h post-infection (Figure 4b).

**Figure 5:**
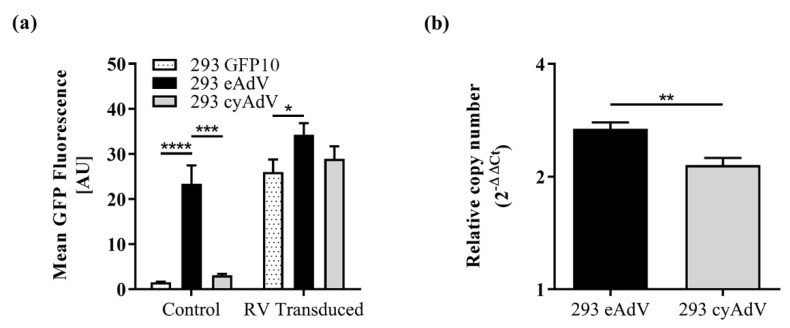
Comparison of GFP10 and GFP11 levels in 293 eAdV and 293 cyAdV sensor cells. (**a**) Parental 293 GFP10 and sensor cells were transduced with a characterized stock of retroviral vectors (RVs) coding for nondistorted GFP11 fragment. After 48 h, control (non-transduced) and transduced cells were analyzed by flow cytometry for mean GFP intensity. Data shown as mean ± SD of three independent experiments. *, *p* < 0.05; ***, *p* < 0.001; ****, *p* < 0.0001, as given by ANOVA followed by Tukey’s post-hoc test. AU, arbitrary units. (**b**) The number of integrated transgene copies per cell (whether coding for eAdV or cyAdV sensors) was determined relatively to 293 GFP10 parental cells by quantitative PCR with primers for *WPRE*, after normalization to *RPL22* reference gene. Data shown as mean ± SD of technical triplicates. **, *p* < 0.01, as given by an unpaired, two-tailed Students’ *t*-test.

In contrast to transient screenings however, eAdV sensor cells showed higher fluorescence emission (Figure 4a). Conversely, cyAdV showed lower fluorescence emission (Figure 4a) and SNR performance (SNR, 1.59 ± 0.07 at 48 h post-infection; Figure 4c). To better elucidate the differences observed between sensor populations, GFP10 and GFP11 levels were analyzed in 293 GFP10 parental cells and eAdV and cyAdV sensor cells. To assess if GFP10 levels were similar and not limiting fragment self-assembly, the two sensor populations were transduced with retrovirus coding for a nondistorted version of GFP11, readily available to self-assemble with GFP10. Upon retroviral transduction, the low background 293 GFP10 parental cells (1.5 ± 0.1 arbitrary units, AU) showed a ~17-fold increase in fluorescence emission (26 ± 2 AU) (Figure 5a). As for both eAdV and cyAdV sensor populations, although with very different background levels (as also observed in Figure 4a), they reached similar fluorescence levels when given nondistorted GFP11 (Figure 5a).

To assess if GFP11 levels were limiting SNR performance of cyAdV sensor cells, transgene copy number integration (relative to 293 GFP10 parental cells) was determined by quantitative PCR. The 293 cyAdV sensor cells showed slightly fewer integrated copies than 293 eAdV sensor cells (Figure 5b). Taken together, these results do not suggest lack of split GFP fragments as the main cause of the observed differences in stable conditions.

Overall, structural distortion of split GFP fragments showed to constitute a valid approach towards the establishment of whole cell biosensing platforms for monitoring of adenoviral infection.

## 4. Discussion

Development of virus-induced cell-based biosensors is essential not only for functional characterization of label-free virus but also critical enzymes—e.g., viral proteases. In that regard, we previously developed cVisensor, a virus-induced biosensor where a circular permuted GFP is cyclized by *Npu* DnaE split intein and the structural distortion maintained until the cleavable sequence is recognized by the AVP [30]. As successive steps of proteolysis and fragment assembly are necessary to reconstitute fluorescence, split GFP-based sensors should theoretically present higher sensitivity and dynamic range than structurally distorted full-length GFP designs.

Herein, we designed and characterized structural distortion strategies of one (single) or both (dual) split GFP fragments to prevent fragment self-assembly before viral proteolysis activation (Scheme 1). Although systems using split GFP for detection of viral proteases were previously reported [15,16], to best of our knowledge, none addressed both structural distortion and conditional proteolytic activation upon virus infection.

Characterization of the three strategies—e11, cy11, and cc10/11—showed indeed lower background fluorescence than cVisensor. However, self-assembly before proteolytic activation was still not completely impaired in single distortion schemes—in particular, cy11 sensors in transient conditions (Figure S3)—as background fluorescence was still detectable. Although GFP10 alone can emit weak fluorescence [37], these results suggest that the herein applied distortions to GFP11 fragment are not sufficient to impair the inherent binding affinity of GFP11 towards GFP10 [14] and, consequently, the irreversible process of chromophore maturation [38]. When in stable conditions, sensor cells with single distortion schemes also presented background fluorescence (Figure 4a). Self-assembly tendency of split GFP fragments is a particular concern in conditions of high fragment expression, as previously observed in split GFP [14] and split yellow fluorescent proteins [39]. Therefore, tight control of the expression levels of both fragments is recommended to avoid unwanted interactions. In addition, one could take further advantage of the use of a tripartite split GFP system [37]. Conversely, cc10/11 dual strategy presented the lowest background fluorescence (Figure 1a, Figure S1 and S4), suggesting efficient impairment of self-assembly when both GFP fragments are structurally distorted. An alternative dual distortion strategy can be envisioned by cyclization of both split GFP fragments. To avoid unwanted intermolecular splicing products however, such as covalent linkage of both GFP fragments before proteolytic activation, usage of orthogonal split inteins is required [40].

When in presence of the target viral proteases—TEVp, AVP, and HIV-1 PR—the majority of the biosensors showed activation of fluorescence emission (Figure 1, Figure 2 and Figure 3), validating their applicability as cell-based switch-on reporters of proteolytic activity. The exception was e11 strategy for the highly specific and active TEVp (Figure 1 and Figure S1). Therein, an unexpected SNR of 0.6 ± 0.2 suggested not only absence of sensor activation, but also loss of background fluorescence. One could hypothesize that cleavage of the e11-ENLYFQS sensors results in an unstable protein, preventing both unspecific (noise) and specific (signal) split GFP assembly. In remarkable contrast, cc10/11 strategy showed an outstanding performance (SNR, 97 ± 21; Figure 1 and Figure S1). This result, using a highly active protease, highlights the full potential of these sensors. Adaptation to the clinically relevant—and less active—AVP and HIV-1 PR showed, in transient screenings, different patterns of sensor performance. While for AVP cy11 strategy showed to be optimal (SNR, 3.5 ± 0.7; Figure 2)—representing a higher SNR performance than cVisensor [30]—for HIV-PR, e11 design was the optimal design (SNR, 6.0 ± 0.6; Figure 3). The lower SNR performances of the clinically relevant proteases might be due to protease activity and cleavage sequence constrains. Regarding the former, while TEVp and HIV-1 PR need no cofactors, AVP is expressed in an almost inactive form. Although active in the cytoplasm [41,42], its maximal activity is reached inside the nucleus in presence of two cofactors: the cleaved 11-residue peptide from the C-terminus of precursor protein VI; and viral DNA [43]. Regarding the latter, in particular for AVP sensors (Figure 2, Figure S2 and S3), different cleavable sequences might be recognized differently by the proteases and also influence biosensor fluorescence emission. Indeed, both sequence length and composition can have tremendous impact on protein structure and function, and potentially interfere with proper folding and topology [44]. As hypothesized, prevalence of predominantly hydrophobic amino acids (e.g., IVGL*G cleavable sequence) leads to a decrease in sensor fluorescence. On the other hand, as EEGE*G has in its composition predominantly hydrophilic amino acids, sensor folding might be less affected by distortion, leading to the observed increase in fluorescence emission. A similar effect was also observed when adding flexible glycine spacers surrounding the cleavable sequence (Figure S3). Taken together, a fine balance between structural distortion levels, cleavable sequence length and composition, and sensor fluorescence (before and after activation) exists, requiring careful optimization and validation for each different target protease. Noteworthy, some non-specific proteolytic cleavage of the sensors may occur when detecting similar proteases or proteases with similar cleavage patterns. While specificity was not assessed in the scope of this work, the developed sensors are envisioned in biotechnological applications evaluating specific viruses in crude viral lysates or purified preparations and not in a mixture of different viruses, as in human diagnostic applications.

After validation of the e11 and cy11 strategies—the top performing strategies for detection of AVP activity—a virus-induced cell-based biosensing platform for detection of label-free adenovirus infection was established. Of notice, the herein stable sensor cells are ‘bulk’ populations and not single cell clones. As such, cell to cell fluorescence variability may arise due to different levels of protein expression and different ratios of GFP10 and GFP11. Still, sensor cells with the e11 strategy allowed live-cell monitoring of infection by fluorescence microscopy and flow cytometry, with fluorescence increasing fast, as soon as 24 h post-infection (Figure 4a,b). Conversely, cy11 sensor cells under-performed (Figure 4c). As seen in Figure 5a, all three populations reached similar and high fluorescence levels when given undistorted GFP11, suggesting similar levels of GFP10 expression. GFP11 levels were indirectly assessed by quantification of copy number integration, since no commercially available primary antibodies for GFP11 exist. As lentivirus-based viral vectors stably integrate the transgene in a non-random fashion, similar integration and expression profiles—in average—for both sensor populations might be expected. Although cyAdV cells showed slightly lower relative number of integrated GFP11 copies (Figure 5b)—and potentially lower levels of protein expression—we hypothesize this difference is not biologically significant to account for the negative impact seen in sensor performance. Other factors should be considered, such as low self-assembly efficiency in stable conditions (due to lower protein expression levels) or cy11 sensor protein degradation. The higher SNR sensor performance observed in transient conditions compared to stable conditions (Figure 2 and Figure 4) may arise from several factors. Although RNA level quantification was not performed to provide insights on these differences, we can hypothesize that transient transfection might lead to higher viral protease and/or sensor protein expression. As the label-free AdVs used in this work can replicate in 293 cells, protease levels during infection should not be a limitative factor. AVP compartmentalization and activation status (as explained above) may, nevertheless, negatively impact sensor activation. In that regard, detection of other cytoplasmatic viral proteases could result in increased sensor activation. On the other hand, increased levels of sensor protein and/or different ratios of GFP10 and GFP11 may be necessary towards a stable sensor platform with improved performance.

Overall, the successful detection of viral infection provides a strong basis for further applicability of these sensor cells in virus detection and quantification, as well in screening of protease inhibitors. Towards that ultimate goal, ‘bulk’ populations—herein characterized for proof-of-concept of our multiple sensor strategies and target proteases—should be cloned, aiming at sensor cells lacking basal activity, with lower cell-to-cell variability, and with increased SNR performance. Finally, the optimal sensing strategy for a specific virus should then be benchmarked against well-established titration protocols. While not expected to compete with the sensitivity of a quantitative PCR assay and be applied in human diagnosis, this label-free assay has the potential to compete with other flow cytometry-based assays, making unnecessary to label the virus.

## 5. Conclusions

Herein, we optimized and characterized cell-based switch-on fluorescent split GFP sensors conditionally activated by viral proteolytic activity. While comparable sensing strategies were only applied in biochemical or bacterial screening assays [15,17], herein we established mammalian cell-based sensors for different cysteine (TEVp and AVP) and aspartic (HIV-1 PR) viral proteases, and a whole cell biosensing platform of adenovirus infection. The split GFP-based sensors have shown an enormous potential, with SNR up to 97. As highlighted by this work nonetheless, the different intrinsic properties of each protease and constraints of cleavable sequence residue composition and length require screening of multiple strategies and extensive sensor optimization. Overall, the limitations and key parameters herein elucidated contribute for the development of better and innovative protease-dependent sensors, whether for protease-containing viruses or cellular proteases.

## Supplementary Materials

The following are available online at https://www.mdpi.com/1424-8220/21/1/24/s1, Table S1: Amino acid residues of the embedded GFP11 (e11) sensor for TEV protease, Table S2: Amino acid residues of the cyclized GFP11 (cy11) sensor for TEV protease, Table S3: Amino acid residues of the coiled-coil GFP10 (cc10) sensor TEV protease, Table S4: Amino acid residues of the coiled-coil GFP11 (cc11) sensor for TEV protease, Table S5: Amino acid sequences of all developed split fluorescent sensors, Table S6: Primers for quantitative PCR, Figure S1: Evaluation of embedment, cyclization, and coiled-coil sensing strategies for detection of tobacco etch virus proteolytic activity, Figure S2: Evaluation of embedded GFP11 (e11) sensor backbones and cleavable sequences for detection of adenoviral proteolytic activity, Figure S3: Evaluation of cyclized GFP11 (cy11) sensor backbones and cleavable sequences for detection of adenoviral proteolytic activity, Figure S4: Evaluation of coiled-coil (cc10/11) strategy for detection of adenoviral proteolytic activity, Figure S5: Evaluation of embedded GFP11 (e11) sensor backbones and cleavable sequences for detection of lentiviral proteolytic activity, Figure S6: Evaluation of cyclized GFP11 (cy11) sensor cleavable sequences for detection of lentiviral proteolytic activity.
