# Supplementary material for: Evaluation of Structurally Distorted Split GFP Fluorescent Sensors for Cell-Based Detection of Viral Proteolytic Activity
Source: Sensors (Basel). 2020 Dec 23;21(1):24. doi: 10.3390/s21010024 (PMC7793068; doi:10.3390/s21010024)
Supplement: Supplementary file 1 [file sensors-21-00024-s001.pdf]

SUPPLEMENTARY DATA

Article

Evaluation of Structurally Distorted Split GFP Fluorescent Sensors for Cell-Based Detection of Viral Proteolytic Activity

Miguel R. Guerreiro [1,2], Ana R. Fernandes [1,2] and Ana S. Coroadinha [1,2,*]

[1] iBET, Instituto de Biologia Experimental e Tecnológica, Apartado 12, 2781-901 Oeiras, Portugal; mguerreiro@ibet.pt (M.R.G.); ana.fernandes@ibet.pt (A.R.F.)

[2] Instituto de Tecnologia Química e Biológica António Xavier, Universidade Nova de Lisboa, Av. da República, 2780-157 Oeiras, Portugal

*** Corresponding Author:**

Dr. Ana Sofia Coroadinha
iBET, Instituto de Biologia Experimental e Tecnológica
Apartado 12, 2781-901 Oeiras, Portugal
Telephone: +351-214469457
E-mail: avalente@ibet.pt

SUPPLEMENTARY EXPERIMENTAL SECTION

Table S1. Amino acid residues of the embedded GFP11 (e11) sensor for TEV protease.

**M T E F G S E L K S W P E V V G K T V D Q A R E Y F T L H Y P Q Y D V Y F L P E G G R D H M V**
**L H E Y V N A A G I T E N L Y F Q S Y N R V R V F Y N P G T N V V N H V P H V G**

Amino acid residues of e11-ENLYFQS sensor are color-coded as follows: in **dark blue**, the 2-41 (TEF…PEG) and 50-71 (YNR…HVG) residues of eglin c; in **grey**, a glycine spacer for GFP11 fragment; in **green**, the GFP11 fragment; in **red**, the ENLYFQS cleavable sequence.

Table S2. Amino acid residues of the cyclized GFP11 (cy11) sensor for TEV protease.

**M I K I A T R K Y L G K Q N V Y D I G V E R D H N F A L K N G F I A S N C F N E N L Y F Q S R D**
**H M V L H E Y V N A A G I T A E Y C L S Y E T E I L T V E Y G L L P I G K I V E K R I E C T V Y S**
**V D N N G N I Y T Q P V A Q W H D R G E Q E V F E Y C L E D G S L I R A T K D H K F M T V D G**
**Q M L P I D E I F E R E L D L M R V D N L P N G G G G S E Q K L I S E E D L**

Amino acid residues of cy11-ENLYFQS sensor are color-coded as follows: in **dark blue**, the C-fragment (IKI…ASN) and N-fragment (CLS…LPN) of *Nostoc punctiforme* DnaE split intein (*Npu* DnaE); in **light blue**, the C-extein (CFN) and N-extein (AEY) residues of *Npu* DnaE; in **green**, the GFP11 fragment; in **red**, the ENLYFQS cleavable sequence; in **grey**, a GGGGS flexible linker; in **black**, the epitope tag derived from c-Myc protein.

Table S3. Amino acid residues of the coiled-coil GFP10 (cc10) sensor TEV protease.

**M E V S A L E K E V S A L E K E V S A L E K E V S A L E K E V S A L E K G S S K G E E L F T G V**
**V P I L V E L D G D V N G H K F S V R G E G E G D A T I G K L T L K F I C T T G K L P V P W P T**
**L V T T L T Y G V Q C F S R Y P D H M K R H D F F K S A M P E G Y V Q E R T I S F K D D G K Y**
**K T R A V V K F E G D T L V N R I E L K G T D F K E D G N I L G H K L E Y N F N S H N V Y I T A**
**D K Q K N G I K A N F T V R H N V E D G S V Q L A D H Y Q Q N T P I G D G P V L L P D N H Y L S**
**T Q T V L S K D P N E K E N L Y F Q S E F G G S K V S A L K E K V S A L K E K V S A L K E K V S**
**A L K E K V S A L K E**

Amino acid residues of cc10-ENLYFQS sensor are color-coded as follows: in **light blue**, E5-coil from E5/K5 heterodimer; in **green**, the GFP10 fragment; in **red**, the ENLYFQS cleavable sequence; in **grey**, GS and EF, residues coded respectively by BamHI and EcoRI endonuclease restriction sites; in **orange**, K5-coil from E5/K5 heterodimer, with amino terminal GGS linker.

Table S4. Amino acid residues of the coiled-coil GFP11 (cc11) sensor for TEV protease.

**M G G S K V S A L K E K V S A L K E K V S A L K E K V S A L K E K V S A L K E R D H M V L H E**
**Y V N A A G I T E N L Y F Q S E V S A L E K E V S A L E K E V S A L E K E V S A L E K E V S A L E**
**K**

Amino acid residues of cc11-ENLYFQS sensor are color-coded as follows: in **orange**, K5-coil from E5/K5 heterodimer, with amino terminal GGS linker; in **green**, the GFP11 fragment; in **red**, the ENLYFQS cleavable sequence; in **light blue**, E5-coil from E5/K5 heterodimer.

Table S5. Amino acid sequences of all developed split fluorescent sensors.

**Name**	**Amino acid sequence**
**Embedded GFP11 strategy (e11)**	
e11-ENLYFQS	En...PEG-G-GFP11-**ENLYFQ*S**-YNR...Ec
e11.v0-LRGAG **(eAdV)**	En...PEG-G-GFP11-**LRGA*G**-YNR...Ec
e11.v0-IVGLG	En...PEG-G-GFP11-**IVGL*G**-YNR...Ec
e11.v0-EEGEG	En...PEG-G-GFP11-**EEGE*G**-YNR...Ec
e11.v1-LRGAG	En...PEG-G-GFP11-G**LRGA*G**G-YNR...Ec
e11.v0-GIFLET	En...PEG-G-GFP11-**GIF*LET**-YNR...Ec
e11.v0-GSGIFLETSL	En...PEG-G-GFP11-**GSGIF*LETSL**-YNR...Ec
e11.v0-IRKILFLDG	En...PEG-G-GFP11-**IRKIL*FLDG**-YNR...Ec
e11.v1-GIFLET	En...PEG-G-GFP11-G**GIF*LET**G-YNR...Ec
e11.v1-GSGIFLETSL	En...PEG-G-GFP11-G**GSGIF*LETSL**G-YNR...Ec
e11.v1-IRKILFLDG	En...PEG-G-GFP11-G**IRKIL*FLDG**G-YNR...Ec
**Cyclized GFP11 strategy (cy11)**	
cy11-ENLYFQS	Dc-**ENLYFQ*S-**GFP11-Dn-myc
cy11.v0-LRGAG	Dc-**LRGA*G-**GFP11-Dn-myc
cy11.v1-LRGAG **(cyAdV)**	Dc-G**LRGA*G**G-GFP11-Dn-myc
cy11.v1-IVGLG	Dc-G**IVGL*G**G-GFP11-Dn-myc
cy11.v1-EEGEG	Dc-G**EEGE*G**G-GFP11-Dn-myc
cy11.v2-LRGAG	Dc-GG**LRGA*G**GG-GFP11-Dn-myc
cy11.v0-GIFLET	Dc-**GIF*LET-**GFP11-Dn-myc
cy11.v0-GSGIFLETSL	Dc-**GSGIF*LETSL-**GFP11-Dn-myc
cy11.v0-IRKILFLDG	Dc-**IRKIL*FLDG-**GFP11-Dn-myc
**Coiled-coil GFP10 and GFP11 strategy (cc10/11)**	
cc10-ENLYFQS	E5-GS-GFP10-**ENLYFQ*S**-EF-K5
cc11-ENLYFQS	K5-GFP11-**ENLYFQ*S**-E5
cc10-LRGAG	E5-GS-GFP10-G**LRGA*G**-EF-K5
cc11-LRGAG	K5-GFP11-G**LRGA*G**-E5

Cleavable sequences in bold, with asterisk representing scissile bond. En…PEG, 1-41 residues of eglin c; GFP11, amino acids coding for GFP11 fragment; YNR…Ec, 50-71 residues of eglin c; Dc, C-fragment of *Nostoc punctiforme* DnaE split intein (*Npu* DnaE) and CFN residues of C-extein; Dn, AEY residues of N-extein and N-fragment of *Npu* DnaE; myc, epitope tag derived from c-Myc protein with a GGGGS flexible linker; E5, E5-coil from E5/K5 heterodimer; GS and EF, residues coded respectively by BamHI and EcoRI endonuclease restriction sites; GFP10, amino acids coding for GFP10 fragment; K5, K5-coil from E5/K5 heterodimer, with amino terminal GGS linker.

Table S6. Primers for quantitative PCR.

**Target gene**	**Primer sequence (5’ → 3’)**
Ribosomal protein L22 (RPL22)	*F*- CTGCCAATTTTGAGCAGTTT
	R- CTTTGCTGTTAGCAACTACGC
Woodchuck Hepatitis Virus Post-Transcriptional Regulatory Element (WPRE)	*F*- ACTGTGTTTGCTGACGCAAC
	R- ACAACACCACGGAATTGTCA

SUPPLEMENTARY RESULTS

(a)

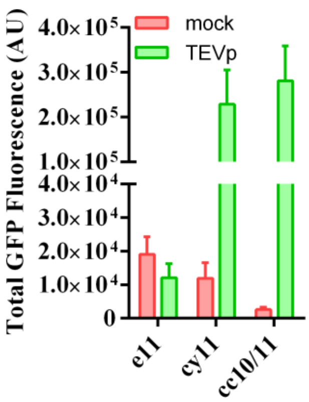

(b)

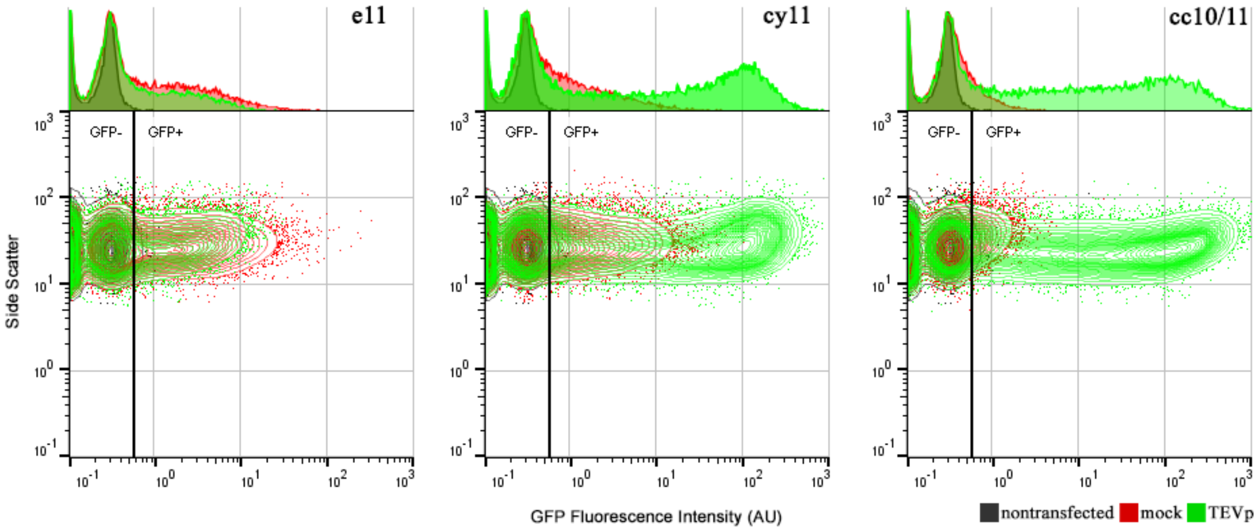

Figure S1. Evaluation of embedment, cyclization, and coiled-coil sensing strategies for detection of tobacco etch virus proteolytic activity. **(a)** 293T cells were transiently co-transfected with plasmids coding for either embedded GFP11 (e11), cyclized GFP11 (cy11) or coiled-coil GFP10 and GFP11 (cc10/11) sensors, GFP10-coding plasmid where needed for complementation, and either a mock plasmid or tobacco etch virus protease (TEVp) coding plasmid. After 48 hours, total GFP fluorescence was measured by flow cytometry. Data shown as mean ± SD of at least three independent experiments. AU, arbitrary units. **(b)** Plots of a representative flow cytometry experiment. Gates were set using non-transfected 293T cells as negative control, and the geometric mean GFP fluorescence intensity of GFP positive cells measured within the positive gate.

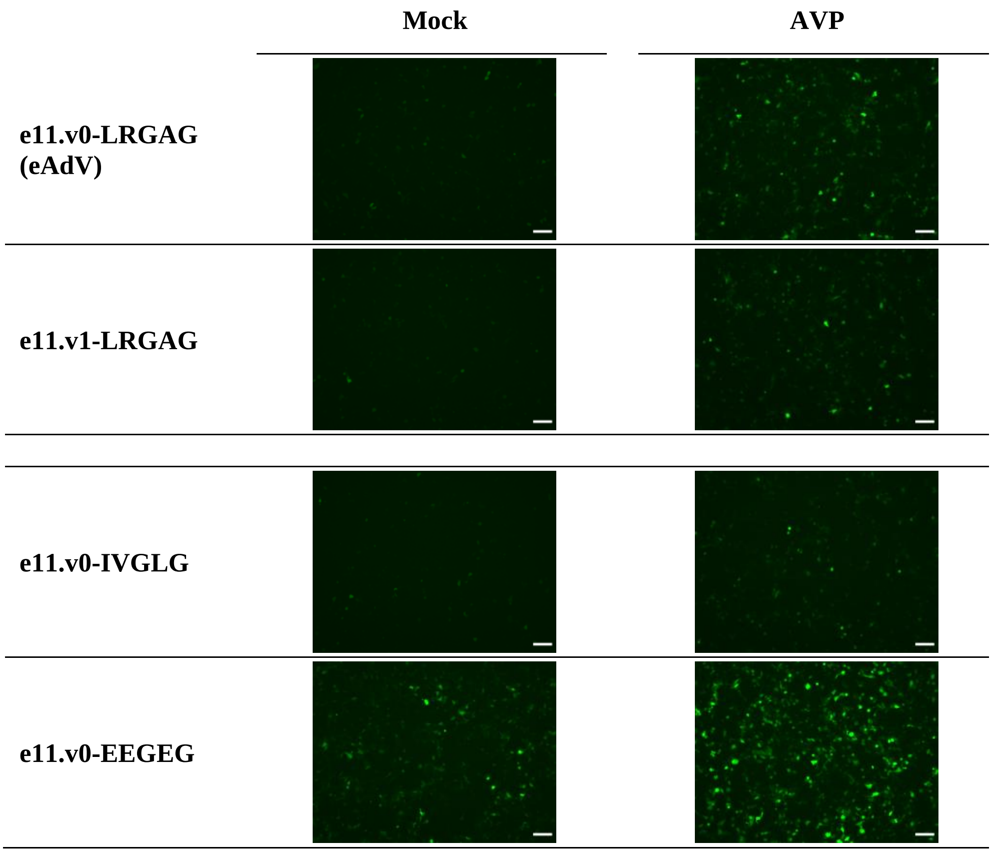

Figure S2. Evaluation of embedded GFP11 (e11) sensor backbones and cleavable sequences for detection of adenoviral proteolytic activity. 293T cells were co-transfected with plasmids coding for one of the different e11 sensors, GFP10 fragment, and either a mock plasmid or adenovirus protease (AVP) coding plasmid. Fluorescence microscopy images were acquired 48 hours post-transfection. Scale bar = 100 µm.

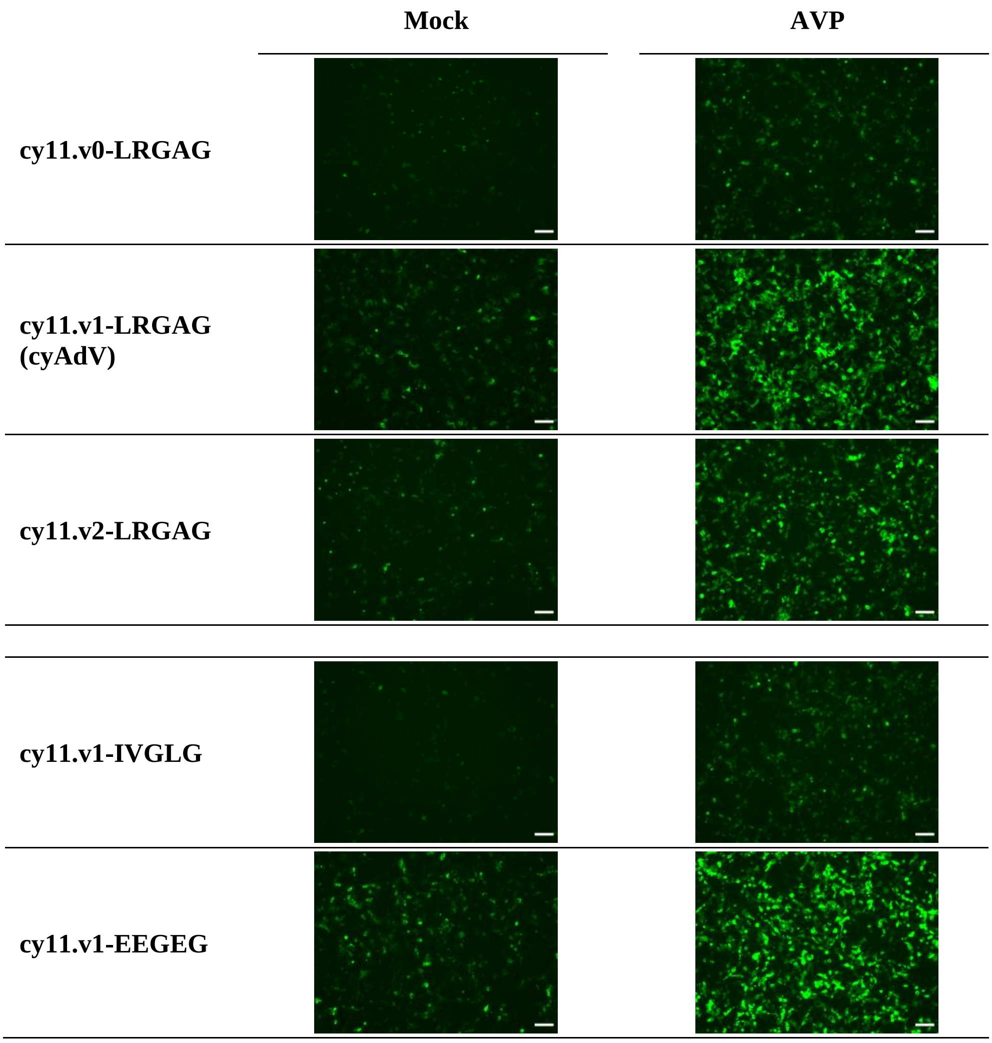

Figure S3. Evaluation of cyclized GFP11 (cy11) sensor backbones and cleavable sequences for detection of adenoviral proteolytic activity. 293T cells were co-transfected with plasmids coding for one of the different cy11 sensors, GFP10 fragment, and either a mock plasmid or adenovirus protease (AVP) coding plasmid. Fluorescence microscopy images were acquired 48 hours post-transfection. Scale bar = 100 µm.

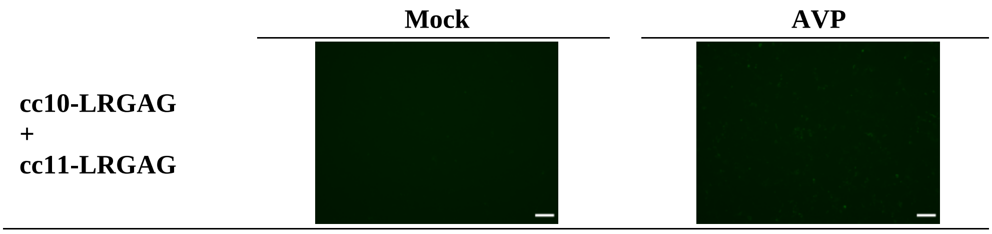

Figure S4. Evaluation of coiled-coil (cc10/11) strategy for detection of adenoviral proteolytic activity. 293T cells were co-transfected with plasmids coding for cc10-LRGAG, cc11-LRGAG, and either a mock plasmid or adenovirus protease (AVP) coding plasmid. Fluorescence microscopy images were acquired 48 hours later. Scale bar = 100 µm.

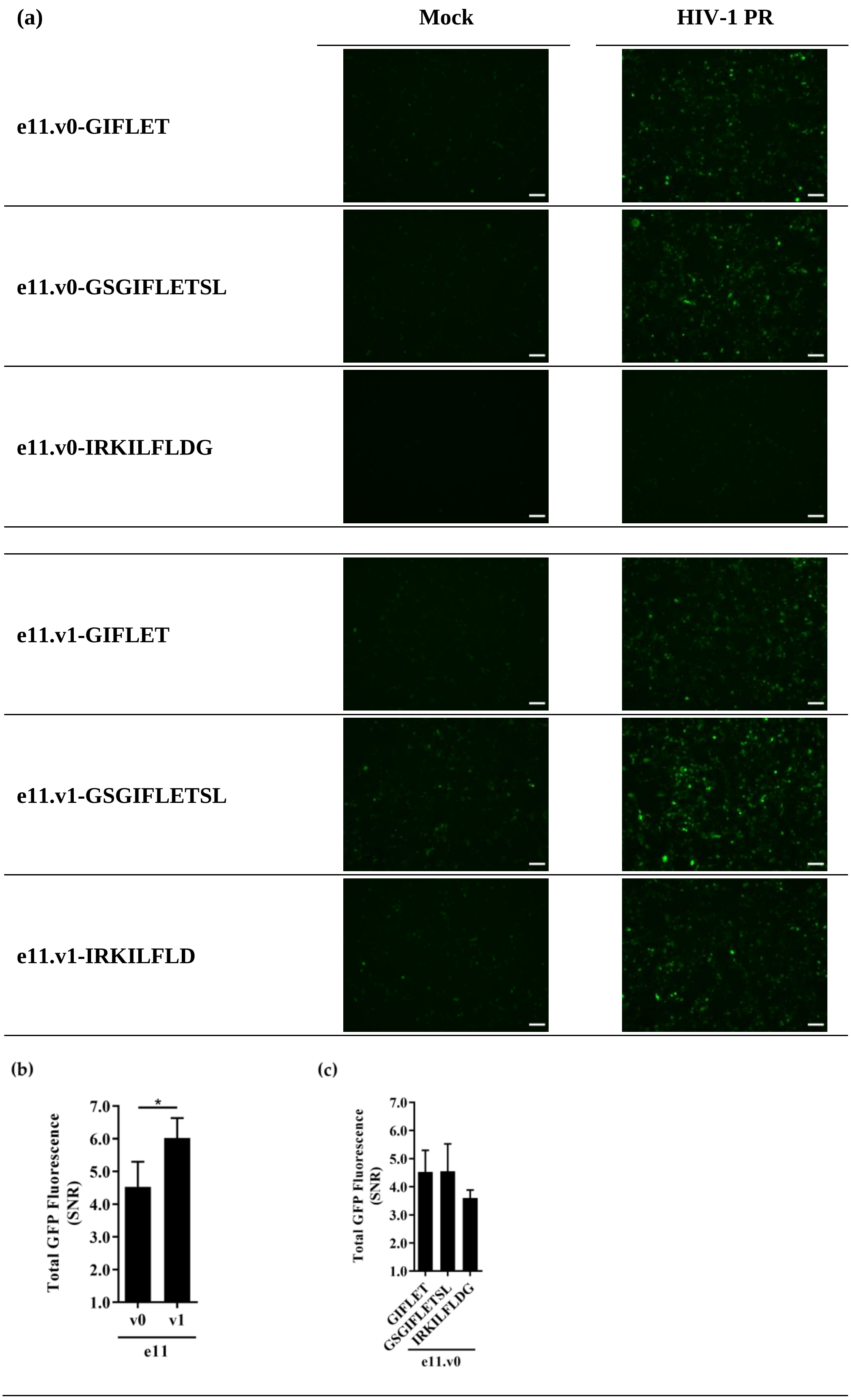

Figure S5. Evaluation of embedded GFP11 (e11) sensor backbones and cleavable sequences for detection of lentiviral proteolytic activity. 293T cells were co-transfected with plasmids coding for one of the e11 sensors, GFP10 fragment, and either a mock plasmid or psPAX2 plasmid (coding for HIV-1 protease, HIV-1 PR). After 48 hours, **(a)** fluorescence microscopy images were acquired and sensor performance of different **(b)** backbones and **(c)** cleavable sequences was assessed by flow cytometry. Data shown as mean ± SD of at least three independent experiments. *, $P < 0.05$; as given by an unpaired, two-tailed Students' t-test. Scale bar = 100 µm.

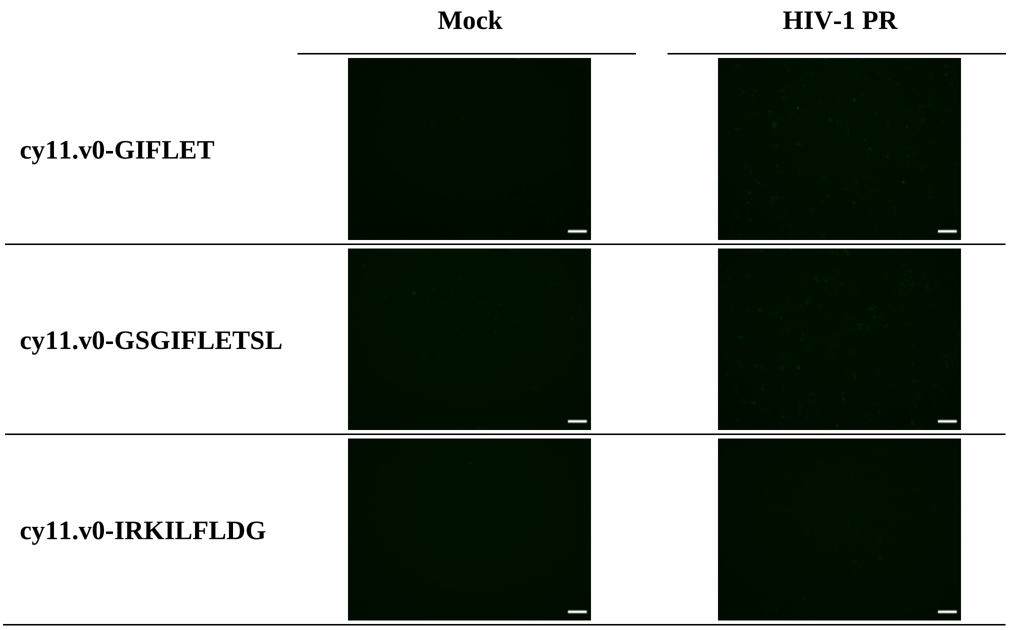

Figure S6. Evaluation of cyclized GFP11 (cy11) sensor cleavable sequences for detection of lentiviral proteolytic activity. 293T cells were co-transfected with plasmids coding for one of the different cy11 sensors, GFP10 fragment, and either a mock plasmid or psPAX2 plasmid (coding for HIV-1 protease, HIV-1 PR). Fluorescence microscopy images were acquired 48 hours post-transfection. Scale bar = 100 µm.
